# Supplementary material for: Patient attitudes towards faecal sampling for gut microbiome studies and clinical care reveal positive engagement and room for improvement
Source: PLoS One. 2021 Apr 8;16(4):e0249405. doi: 10.1371/journal.pone.0249405 (PMC8031379; doi:10.1371/journal.pone.0249405)
Supplement: S1 File — Additional information on statistical analyses and results of the interview conducted in the general population cohort. (DOCX) [file pone.0249405.s003.docx]

**S1 File. Supplementary Methods**

**Statistical analyses**

Descriptive statistics were determined for each question using R^14^ (**S1 Table**). Chi-Square tests and Fisher’s exact tests were performed to determine statistically significant differences between counts. The following five associations were calculated:

1. Willingness to collect faecal samples for future screening and care vs. GI-disease (Fisher’s exact test): (Q36 answer A (willing to both collect and freeze) + Q36 answer B (willing to collect but not freeze)) vs. not willing to collect faecal samples for future screening and care (Q36 answer C (neither willing to collect nor freeze), between patients of the gastrointestinal disease cohorts (IBD-Willing + IBD Unwilling + Participants without an Identification number) and patients not affected by a gastrointestinal disorder (SLE + Sjögren + Oncology + No Identification number).
2. Willingness to collect faecal samples for future screening and care vs. Home situation (Fisher’s exact test): Patients living alone (Q4 answer A (living alone) vs. Patients living with cohabitants (Q4 answer B (with partner) + Q4 answer C (with partner and child), + Q4 answer D (only with child) + Q4 answer E (with parents) + Q4 answer F (with roommates)).
3. Willingness to collect faecal samples for future screening and care vs. Clarity of the instruction manual (Fisher’s exact test): Clarity of the instruction manual (Q17 answer A (very clear) vs. Q17 answer B (clear) vs. Q17 answer C (neither clear nor unclear) vs. Q17 answer D (unclear) vs. Q17 answer E (very unclear).
4. Willingness to collect faecal samples for future screening and care vs. Clarity of oral instruction (Fisher’s exact test): Q16 with same answer options as Q17.
5. Willingness to store faecal samples in the home freezer for future screening and care vs. Knowing the purpose of freezing the samples (Chi-Square test of independence with Yate’s continuity correction): Willingness to store faecal samples in the home freezer for future screening and care (Q36 answer A (willing to collect and freeze)) vs. not willing to store faecal samples in the home freezer for future screening and care (Q36 answer B (willing to collect but not freeze) + Q36 answer C (neither willing to collect nor freeze)); patients who know the purpose of freezing (Q33 answer A (know purpose of freezing)) and patients who do not know the purpose of freezing (Q33 answer B (did not know purpose of freezing)).

**Results of the Interview with Healthy Volunteers**

Interviews were conducted between August 26 and November 11, 2015. Aggregated responses were provided to the authors. For the research in question, interviewees had been provided with five tubes to be filled with faecal matter. Most participants (473 of 478, 99.0%) were able to fill all tubes. Five participants failed to do so because it was either too inconvenient (n=1), they had cut the tube incorrectly (n=1), the pipette broke (n=1), the tube broke (n=1) or only three tubes were provided (n=1). Of these five participants, two wanted to retry and requested new material. Overall, nine participants (1.9%) viewed the faecal sample collection as “very inconvenient”, 25 (5.2%) as “slightly inconvenient”, 68 (14.2%) had a neutral attitude, 137 (28.7%) found it “not inconvenient”, and 238 (49.8%) viewed it as “not inconvenient at all".

Most interviewees (457 of 478, 95.6%) indicated they would be willing to participate in similar research if asked again in future. Those who indicated that they would not like to participate again (17 of 478, 3.6%) reported that it was a hassle to fill all tubes (n=3), their partner found it inconvenient (n=1), they found it a distasteful procedure (n=2), or that it was too time consuming (n=1). The 10 participants who indicated they were not yet certain if they would participate in the future indicated their decision would depend on future circumstances related to time, work, and disease.
