# Supplementary material for: Patient attitudes towards faecal sampling for gut microbiome studies and clinical care reveal positive engagement and room for improvement
Source: PLoS One. 2021 Apr 8;16(4):e0249405. doi: 10.1371/journal.pone.0249405 (PMC8031379; doi:10.1371/journal.pone.0249405)
Supplement: Attachment — Submitted filename: Response_to_reviewers_ed1.docx [file pone.0249405.s004.docx]

**Reviewer 1**

**Q.1.1:** Thank you for writing such a clear and interesting article on the patient perspective towards stool collection. I have a few minor comments.

Introduction, Paragraph 1: Authors state ‘little is known about participant perspectives on collecting faecal samples for microbiome research and future care, with available literature currently limited to several studies examining participant experiences with the faecal occult blood test (FOBT) used in colorectal cancer screening, the results of which mainly capture experiences coloured by the fear of having cancer’ and reference 2 other studies. I agree that literature on this topic is limited however it is not only limited to FOBT studies. The Lecky et al study for example examined patients both with and without previous experience of providing a stool sample and does not state that recruited participants had collected samples for FOBT. Other work in this arena, not just linked to FOBT, also include:

McNulty, C.A., Lasseter, G., Newby, K. et al. Stool submission by general practitioners in SW England - when, why and how? A qualitative study. BMC Fam Pract 2012, 13:77.

McSweeney B, Allegretti JR, Fischer, M et al. In search of stool donors: a multicenter study of prior knowledge, perceptions, motivators, and deterrents among potential donors for fecal microbiota transplantation, Gut Microbes 2020, 11:1, 51-62.

Please make this more clear in your introduction.

**R.1.1:** We thank the reviewer for the positive feedback and suggestions to improve our manuscript. We agree that our findings connect well with other disciplines such as the diagnosis of intestinal infectious diseases as part of clinical routine, or the search for stool donors for fecal microbiota transplantation. We now refer to these articles in the introduction.

**Page 3, lines 9-12:**

“*Moreover, as the clinical interest grows in the use of fecal microbiota transplantation (FMT) for dysbiosis-related disorders such as recurrent Clostridium difficile associated diarrhea and IBD, so will the need for voluntary stool donors.[10–12]”*

[10] McSweeney B, Allegretti JR, Fischer M, Xu H, Goodman KJ, Monaghan T, et al. In search of

stool donors: a multicenter study of prior knowledge, perceptions, motivators, and deterrents

among potential donors for fecal microbiota transplantation. Gut Microbes 2019; 11: 51-62.

[11] Moayyedi P, Surette MG, Kim PT, Libertucci J, Wolfe M, Onischi C, et al. Fecal Microbiota

Transplantation Induces Remission in Patients With Active Ulcerative Colitis in a Randomized

Controlled Trial. Gastroenterology 2015; 149: 102-109.e6.

[12] Vrieze A, Nood EV, Holleman F, Salojärvi J, Kootte RS, Bartelsman JFWM, et al. Transfer of

Intestinal Microbiota From Lean Donors Increases Insulin Sensitivity in Individuals With

Metabolic Syndrome. Gastroenterology 2012; 143: 913-916.e7.

**Page 3, lines 18-24:**

*“Other studies report patient barriers to fecal sample collection in general practice, including difficulty with the process, embarrassment and concerns around hygiene. [14, 15] Despite these barriers, most patients do provide fecal samples in the clinical setting, because they are unwell and have been recommended to do so. [15] While personal gain has been identified as the main motive for collecting and returning a stool sample in clinical care,[14] there is no direct personal benefit for voluntary stool donors for FMT or microbiome research, who may face similar barriers.”*

[14] Lecky DM, Hawking MK, McNulty CA. Patients’ perspectives on providing a stool sample to

their GP: a qualitative study. Br J Gen Pract 2014; 64: e684–e693.

[15] McNulty CA, Lasseter G, Newby K, Joshi P, Yoxall H, Kumaran K, et al. Stool submission by

general practitioners in SW England - when, why and how? A qualitative study. BMC Family

Practice 2012; 13: 77.

**Q.1.2:** Discussion paragraphs 2 and 3: These paragraphs are more suited to introducing the topic and the need for stool collection rather than discussing the findings of this study and its relevance and/or implications**.**

**R.1.2:** We agree with the reviewer that the need for stool collection is more suitable to introduce the topic and have moved these paragraphs to the introduction.

**Page 3, lines 5-12:**

“*The results of these studies hold great promise for clinical applications that include the use of microbiome features as diagnostic biomarkers,[5] determinants of disease activity,[3] and predictors of individual drug response.[6,7] The microbiome itself may also be a treatment target for prebiotic, probiotic, antibiotic and dietary interventions. [2,8,9] Moreover, as clinical interest grows in the use of fecal microbiota transplantation (FMT) for dysbiosis-related disorders such as recurrent Clostridium difficile infection and IBD, so will the need for voluntary donors.[10–12]”*

**Q.1.3:** As previously mentioned, other research has been carried out in these areas therefore the discussion would benefit from more comparison to this literature highlighting similarities and differences and potential reasons for this.

**R.1.3:** We agree with the reviewer and added a comparison of the identified attitudes towards fecal sampling for gut microbiome research in our study with previous reports on the perspectives on collecting or donating fecal samples for clinical care or FMT.

**Page 12, lines 16-19:**

*“This is in line with a previous report of the attitudes of 400 patients towards participation in clinical trials conducted at an internal medicine ward.[25] Positive feedback on how FMT can help patients were also motivators to donate fecal samples for FMT.[10]”*

**Page 13, line 7-13:**

*“McSweeney et al. also identified altruism as the main motive to donate fecal samples for FMT and many patients who were willing to donate fecal samples said to do so to help those who were ill and contribute to progress in scientific research. [10] As expected, this differs from the motives of patients who collect or receive fecal samples as part of their clinical care, where personal gain is the main incentive. [14]* *Despite concerns around hygiene, logistics and privacy, most patients return their faecal sample to their doctor because they were recommended to do so for their own health.[15]”*

**Page 15, line 5-12:**

*“Other studies identified barriers towards fecal sampling in clinical care, including difficulty with the collection process, lack of information given by doctors and inability to return the sample to the institution. [14, 15] The difficulty of collecting the fecal sample was one of the major factors impacting FOBT response in a South African study. [29] Higher donation frequency, logistics of collection or transport of fecal samples, screening process, lack of public awareness and negative social perception were identified as deterrents to donate stool for FMT. [10]”*

**Page 15, line 20 – Page 16, line 2:**

“*Similar to our findings, studies indicate that patients collecting fecal samples for clinical care value an information leaflet provided with the stool collection kit [14] and that screening compliance for the FOBT is significantly improved when patients obtain this information. [30]*”

[10] McSweeney B, Allegretti JR, Fischer M, Xu H, Goodman KJ, Monaghan T, et al. In search of

stool donors: a multicenter study of prior knowledge, perceptions, motivators, and deterrents

among potential donors for fecal microbiota transplantation. Gut Microbes 2019; 11:51-62.

[14] Lecky DM, Hawking MK, McNulty CA. Patients’ perspectives on providing a stool sample to

their GP: a qualitative study. Br J Gen Pract 2014; 64: e684–e693.

[15] McNulty CA, Lasseter G, Newby K, Joshi P, Yoxall H, Kumaran K, et al. Stool submission by

general practitioners in SW England - when, why and how? A qualitative study. BMC Family

Practice 2012; 13: 77.

[29] Price CL, Szczepura AK, Gumber AK, Patnik J. Comparison of breast and bowel cancer

screening uptake patterns in a common cohort of South Asian women in England. BMC Health

Services Research 2010; 10: 103.

[30] O’Carroll RE, Steele RJ, Libby G, Brownlee L, Chambers JA. Anticipated regret to increase

uptake of colorectal cancer screening in Scotland (ARTICS): study protocol for a randomised

controlled trial. BMC Public Health 2013; 13: 849.

**Reviewer 2**

**Q.2.1:** The topic of the manuscript is interesting and important to tell researchers who are going to collect stool samples. This study highlighted the difficulty for achieving stool sample for microbial study. The “dirty” is one of the important factors affecting the attitudes to response the survey and collect stool sample. The lower response rate for this survey is the main limitation of this study, which might be the bias of the survey. However, authors did not clearly state this difficulty in INTRODUCTION or discuss this point in DISCUSSION. As I know, the similar survey on patients’ or physicians’ attitude to process fecal transplant have the similar difficulty in practice. These should be discussed for supporting authors’ findings. The following reports should be included for discussion:

Patients' views on fecal microbiota transplantation: an acceptable therapeutic option in inflammatory bowel disease? Eur J Gastroenterol Hepatol 2017.

Perceptions of fecal microbiota transplantation for Clostridium difficile infection: factors that predict acceptance. Ann Gastroenterol. 2017.

The recognition and attitudes of postgraduate medical students toward fecal microbiota transplantation: a questionnaire study. Therap Adv Gastroenterol. 2019

**R.2.1:** We thank the reviewer for the thorough inspection of our manuscript. We agree that the non-response bias should be stated more clearly as a limitation in the discussion section. Moreover, we agree that the above-mentioned literature should be described and compared to our results more elaborately in the discussion.

Therefore, we have largely rewritten our discussion including the literature and limitations mentioned by the reviewer.

**Page 11, line 15 – Page 12, line 11:**

“*Other studies assessed patient willingness to receive or donate stool samples for FMT, the transfer of fecal material containing microbiota from a healthy donor into a diseases patient. One study found that 77% of patients visiting the gastroenterologist would undergo FMT if medically indicated. [22] Conversely, only 36.9% of IBD patients were willing to undergo FMT in a report by Zeitz et al. [23] Familiarity with the gut microbiome might contribute to a higher willingness to participate in gut microbiome research in our study compared to FMT. Only 46.5% of IBD patients [23] and 12% of patients visiting a gastroenterologist [22] knew about FMT. Interestingly, the willingness rate of IBD patients to undergo FMT almost doubled after an information leaflet was provided. [23] Recognition of FMT in postgraduate medical students was equally low. [24] While nearly half of the students had not heard about FMT, the majority recognized that disrupting and restoring gut microbiota played an important role in the pathogenesis and prevention of diseases. In the same study, willingness to undergo FMT or donate samples was significantly higher among those who were familiar with FMT. [24]*”

[22]	Park et al. Perceptions of fecal microbiota transplantation for Clostridium difficile infection: factors that predict acceptance. Ann Gastroenterol 2017; 30: 83–88.

[23]	Zeitz et al. Patients’ views on fecal microbiota transplantation: an acceptable therapeutic option in inflammatory bowel disease? Eur J Gastroenterol Hepatol 2017; 29: 322–330.

[24] Wu et al. The recognition and attitudes of postgraduate medical students toward fecal

microbiota transplantation: a questionnaire study. Therap Adv Gastroenterol 2019; 12:

1756284819869144.

**Page 13, lines 14-20:**

“*Similarly, studies showed that the majority of patients would undergo FMT if it was medically indicated and recommended by their doctor [22] and willingness to undergo FMT was positively associated with disease severity and previous TNF-treatment in IBD patients. [23, 28] Naturally, recommendation by your own doctor is not part of when recruiting volunteers for microbiome research or healthy donors for FMT, making it even more crucial to inform volunteers about the process and remove any barriers in order to obtain sufficient sample sizes or to guarantee cost-effectiveness.*”

[28]	Kahn et al. Fecal bacteriotherapy for ulcerative colitis: patients are ready, are we? Inflamm Bowel Dis 2012; 18: 676–684.

**Page 14, lines 11-19:**

“*Fear of infectious diseases and disgust about the procedure were also identified as the most common concerns of patients to undergo FMT. [22, 23] This is underlined by the finding that IBD patients would choose a colonoscopy as the preferred route of FMT rather than an enema or nasogastric tube. [22, 23] Even post-graduate medical students considered donating feces troublesome because it hampered their privacy and also showed concerns about the acceptability among patients. [24] Privacy was not a big concern in our study. While screening for FMT requires the donor to provide a lot of private information as not all stool samples are suitable, participants in gut microbiome research might feel more anonymous.*”

**Page 15, lines 20-22:**

“*This may be particularly important in populations with a lower health illiteracy, with a study showing that higher education levels are strong predictors of FMT acceptance in patients. [22]*”

**Page 16, lines 7-11:**

“*Another study even showed that having children and being married were strong predictors of FMT acceptance in patients. [22] It is possible that patients with children are more likely to embrace FMT, even though it may be unappealing in nature, because of their responsibility towards their family. [27]*”

[27] Prainsack and Buyx. A solidarity-based approach to the governance of research

biobanks. Medical Law Review 2013; 21: 71–91.

**Page 16, line 12 – Page 17, line 5:**

*“Strengths and limitations*

*Our questionnaire study was limited by knowing only the answers of the respondents. IBD patients who previously disagreed to participate in our gut microbiome studies (IBD-Unwilling, n=83) were also less likely to respond to the questionnaire (n=3), making it difficult to assess their reasons to refuse participation. Overall, the 39.1% response rate to our questionnaire is in line with the recognised average response rate for postal surveys (40%). [31] Another survey of IBD patients to investigate their perspectives on FMT, obtained a response rate of 31.4%. [23] The positive attitudes towards fecal sample collection in our study may not always be representative of other patients, and attitudes may differ depending on the reason for stool sample collection, e.g. samples collected for research vs. those collected for diagnosis of a potential disease (a process that may be accompanied by fear), or the health care setting, e.g. secondary vs. routine primary care. The strength of our study is that we were able to obtain information on the attitudes to, motives for and barriers to participation in gut microbiome research of 780 patients with different disorders and healthy volunteers, which has not been assessed to date. We obtained enough information to formulate the following conclusions and recommendations for both gut microbiome researchers and clinicians.*”

**Q.2.2:** Minor comments as below: In introduction, microbiota is a target for fecal microbiota transplantation too. This is very important. Please add it in the sentence.

**R.2.2:** We agree with the reviewer. The other reviewer has also addressed this issue (Q.1.1). We have now added the prospect of fecal transplantation in the introduction.

**Page 3, lines 9-12:**

“*Moreover, as clinical interest grows in the use of fecal microbiota transplantation (FMT) for dysbiosis-related disorders such as recurrent Clostridium difficile infection (CDI) and IBD, so will the need for voluntary donors.”*

**Q.2.3:** Methods - There were nearly 40 questions in your questionnaire. Why do you only calculate the five questions which you mentioned?

**R.2.3:** For all factors assessed by the questionnaire (38 questions), we calculated the average (SD) and counts (%), respectively (S1 Table). This way, an overview of the participants’ answers on all the assessed factors was displayed, including views on the participants’ home situation, whether they felt free to refuse participation, their intrinsic motivation to collect feces, and logistic aspects such as type of toilet, freezer and time they had to wait until the sample was picked up.

After presenting these descriptive statistics of the participants’ perspectives on the fecal sample collection, we wanted to answer a limited number of specific questions that we think would help microbiome researchers conduct better studies, rather than perform statistical analyses comparing every question to every factor in order to find any statistically significant differences.

**Q.2.4:** In table 1, the number of patients in “Maximum time patients want to store fecal samples in their freezer” should subtract 73 as shown in “Unpleasant to store fecal samples in home freezer?”. Seventy-three patients are unpleasant to store fecal samples in home freezer. So they are not necessary to consider the time to store fecal sample.

**R.2.4:** We understand the remark of the reviewer and have subtracted these 73 participants from this equation in the results, Table 1 and Supplementary Table 1.

**Page 8, line 9 – 11:**

*“However, while most patients were willing to store a stool sample in their freezer, many were only willing to do so for a brief period of time: maximum 1 to 3 days (14.6%), 1 week (25.3%), or 2 to 4 weeks (11.1%). 40.9% said that they did not mind storing faecal samples for a longer time.”*

**Table 1 (page 10)**

| ***Maximum time patients want to store faecal samples in their freezer*** |  | ***All patients*** |  |  |
| --- | --- | --- | --- | --- |
|  | *I do not want that* | *2 (0.9%)* |  |  |
|  | *1 to 3 days* | *33 (14.6%)* |  |  |
|  | *1 week* | *57 (25.3%)* |  |  |
|  | *2 to 4 weeks* | *25 (11.1%)* |  |  |
|  | *>1 month* | *6 (2.7%)* |  |  |
|  | *I do not mind* | *92 (40.9%)* |  |  |
|  | *No answer* | *10 (4.4%)* |  |  |
|  | *Total* | *225 (100%)* |  |  |

**Q.2.5:** Results - 97.4% had collected a faecal sample for prior gut microbiome research projects. Is this data for question eight in the Supplementary Table 1? An error?

**R.2.5:** Indeed, 97.4% had collected a faecal sample for gut microbiome research before (summing up answers A to D in question 8 “Did you ever participate in research for which you had to collect and deep-freeze a small stool sample?”: A) yes, 1 time + B) yes, 2-5 times + C) yes, 6-10 times + D) yes, >10 times). Only in the IBD cohort we were also able to send questionnaires to patients who previously refused to participate in gut microbiome studies, of which 3 patients responded (IBD-Unwilling, n=3). The other cohorts (melanoma, Sjögren’s syndrome, SLE) were comprised of gut microbiome research participants. We agree that this needs a better explanation in the methods section.

**Page 4, lines 17-30:**

*A questionnaire (****S1 Table****) was sent in January 2017 to 772 patients who had previously been recruited at the University Medical Center Groningen in the Netherland for gut microbiome studies for which they needed to provide a faecal sample. These patients had been included in four disease-specific cohorts: IBD (n=660), melanoma (n=9), Sjögren’s syndrome (n=55) and systemic lupus erythematosus (SLE) (n=48) (****Fig 1****). The latter three cohorts only comprised participants who joined the gut microbiome studies. The questionnaire was aimed at obtaining their experiences and identifying barriers encountered during the collection process. With the IBD cohort, we were also able to send out questionnaires to patients who previously refused to participate in gut microbiome research. Questionnaire recipients in the IBD cohort therefore comprised both patients previously willing to collect a stool sample for research (n=577, IBD-Willing) and patients previously not willing to do so (n=83, IBD-Unwilling), indicating a willingness rate of 87.4% of the IBD microbiome study prior to this survey.”*

We also asked if patients were willing to participate again *after* their experiences.

**Page 11, lines 10-14**:

“*Our study has demonstrated that stool sample collection for gut microbiome studies and future clinical applications is acceptable to the majority of IBD patients and even to population controls.* *Most IBD patients (87.4%) were willing to participate in our previous stool sample collection (IBD-Willing, n = 577) and most respondents (82.9%) and interviewees (95.6%) indicated that they were willing to participate again.”*

**Q.2.6:** Results - According to your definition in the Supplementary Method, the patients with gastrointestinal disease includes “IBD willing + IBD unwilling + no identification number” participants. You mentioned in the results that 250 patients had GI-disorder. This may be a statistical error. In addition, this is a little different from the data in the questionnaire. Because you asked participants in question eight if they had been diagnosed with intestinal disease, and only 42 patients answered no.

**R.2.6:** We thank the reviewer for his remark. This calculation was indeed performed based on the participants belonging to the IBD cohort. The reviewer is referring to the answers to question 6 in the questionnaire (**S1 Table**), where we asked the participants specifically about the presence of any intestinal diseases. Indeed, this data is different from the definition by cohort (IBD cohort vs. No IBD cohort). We have now also performed the analysis using question 6 to define presence or absence of intestinal disease: There was no correlation between GI-disease (Q6) and willing to participate in future faecal sample collection (p = 0.564, **Table 1**).

**Table 1 (page 9):**

| ***Willing to collect faecal samples for future healthcare***  ***Split by IBD / No IBD*** | ***Willing to collect*** | ***IBD cohort*** | ***Non-IBD***  ***cohort*** |  |
| --- | --- | --- | --- | --- |
|  | *Yes* | *224 (89.6%)* | *43 (89.6%)* | *P = 0.673* |
|  | *No* | *15 (6.0%)* | *4 (8.3%)* |  |
|  | *Did not fill in* | *11 (4.4%)* | *1 (2.1%)* |  |
|  | *Total* | *250 (100%)* | *48 (100%)* |  |
| ***Willing to collect faecal samples for future healthcare*** | ***Willing to collect*** | ***GI-disorder*** | ***No GI-disorder*** |  |
| ***Split by GI-disorder / No GI-disorder*** | *Yes* | *205 (81.0%)* | *38 (90.5%)* |  |
|  | *No* | *34 (13.4%)* | *4 (9.5%)* | *P = 0.564* |
|  | *Did not fill in* | *14 (5.5%)* | *0 (0.0%)* |  |
|  | *Total* | *253 (100%)* | *42 (100%)* |  |

**Q2.7:** Limitation should better not be included in conclusion. Please put it to the discussion.

***R2.7:*** The limitations now appear at the end of the discussion on page 16, lines 1-13.

**Q2.8:** Conclusions include too much content. Please try to use summative words in this part and put the current content to suitable part.

***R.2.8:*** The conclusion has been adapted. **Page 25, lines 7-21**:

*“Targeting the gut microbiome will soon be part of the diagnostic process and treatment of IBD and other diseases that are associated with microbial dysbiosis [5,6,31,32] requiring repeated sampling from patients.[33] Here, we assessed the perspectives of patients and healthy volunteers on faecal sampling for gut microbiome research. We derive the following recommendations for gut microbiome researchers and clinicians:*

(1) *Gut microbiome researchers setting up new cohorts or clinicians trying new faecal tests should not shy away from doing so.*
(2) *Gut microbiome researchers and clinicians should explain why their collection protocol was designed in a specific way.*
(3) *In studies where a time-series of many stool samples needs to be collected, researchers should consider providing participants with a small freezer.*
(4) *Researchers and clinicians should inform participating patients and healthy volunteers about the outcome of the research.”*

**Q.2.9:** The structure of the manuscript is unconventional. Please refer the regular journal style.

***R.2.8:*** We have changed the structure of our manuscript accordingly.
